# A novel Bi_4_Ti_3_O_12_/Ag_3_PO_4_ heterojunction photocatalyst with enhanced photocatalytic performance

**DOI:** 10.1186/s11671-017-2377-1

**Published:** 2017-11-28

**Authors:** Chengxiang Zheng, Hua Yang, Ziming Cui, Haimin Zhang, Xiangxian Wang

**Affiliations:** 1 State Key Laboratory of Advanced Processing and Recycling of Non-ferrous Metals, Lanzhou University of Technology, Lanzhou, 730050 China; 2 School of Science, Lanzhou University of Technology, Lanzhou, 730050 China

**Keywords:** Bi_4_Ti_3_O_12_ nanoparticles, Ag_3_PO_4_ nanoparticles, Bi_4_Ti_3_O_12_/Ag_3_PO_4_ heterojunction, Photocatalytic performance, Photocatalytic mechanism

## Abstract

In this work, we integrated Ag_3_PO_4_ with Bi_4_Ti_3_O_12_ to form Bi_4_Ti_3_O_12_/Ag_3_PO_4_ heterojunction nanocomposites by an ion-exchange method. The as-prepared Bi_4_Ti_3_O_12_/Ag_3_PO_4_ composites were systematically characterized by means of XRD, SEM, TEM, BET, XPS, UV-vis DRS, EIS, PL spectroscopy, and photocurrent response. SEM, TEM, and XPS results demonstrate the creation of Bi_4_Ti_3_O_12_/Ag_3_PO_4_ heterojunction with obvious interfacial interaction between Bi_4_Ti_3_O_12_ and Ag_3_PO_4_. PL spectra, EIS spectra, and photocurrent responses reveal that the composites display an enhanced separation efficiency of photogenerated electron-hole pairs, which is due to the charge transfer between Bi_4_Ti_3_O_12_ and Ag_3_PO_4_. Rhodamine B (RhB) was chosen as the target organic pollutant to evaluate its degradation behavior over Bi_4_Ti_3_O_12_/Ag_3_PO_4_ composites under simulated sunlight irradiation. Compared to bare Bi_4_Ti_3_O_12_ and Ag_3_PO_4_ nanoparticles, the composites exhibit a significantly enhanced photocatalytic activity. The highest photocatalytic activity is observed for the 10% Bi_4_Ti_3_O_12_/Ag_3_PO_4_ composite with 10% Bi_4_Ti_3_O_12_ content, which is about 2.6 times higher than that of bare Ag_3_PO_4_. The photocatalytic mechanism involved was investigated and discussed in detail.

## Background

With the rapid development of economy, environmental pollution has become one of the most serious problems for human. Particularly, a huge amount of wastewater containing various organic dyes and pigments has been generated annually from chemical industries like paper, textile, paint, and cosmetic manufacturers all over the world. Before releasing the wastewater into the river, those organic pollutants must be removed since most of them are non-biodegradable and carcinogenic and pose an immense threat to the environment and human health. Semiconductor photocatalysis has been received as one of the most promising wastewater treatment technologies [1–9]. This technology can utilize solar energy as the power source for the organic pollutant decomposition. To utilize the solar energy more effectively in the photocatalysis, it is highly desirable to develop visible-light-responsive photocatalysts since visible light accounts for 45% of the solar energy. Moreover, to achieve a good photocatalytic activity of the photocatalyst, the photogenerated electron-hole (e^−^-h^+^) pairs must be effectively separated because the photocatalytic reaction is associated with the photogenerated electrons and holes [10, 11].

Recently, silver orthophosphate (Ag_3_PO_4_) with a bandgap energy of ~ 2.4 eV has been extensively studied as a promising visible light photocatalyst [12–31]. First principle calculations based on the density functional theory suggested that Ag_3_PO_4_ has a highly dispersive band structure of the conduction band minimum resulting from Ag *s*-Ag *s* hybridization without localized *d* states [32], which is advantageous for the separation of electron hole pairs as well as the electron transfer. Furthermore, Ag_3_PO_4_ has a quantum efficiency much higher than the values reported for other semiconductors (~ 90% at λ > 420 nm) [12, 13]. These make Ag_3_PO_4_ an excellent photocatalytic activity for the decomposition of organic pollutants as well as O_2_ evolution from water splitting under visible light irradiation. However, there are still some limitations in the Ag_3_PO_4_ photocatalyst. It is noted that the conduction band potential of Ag_3_PO_4_ is more positive than that of the hydrogen electrode [12]. This means that if there are no sacrificial electron acceptors involved in the photocatalytic system, the photogenerated electrons could reduce the lattice Ag^+^ in Ag_3_PO_4_ into metallic Ag during the photocatalytic process. This self-photocorrosive phenomenon leads to a decrease in the photocatalytic stability of Ag_3_PO_4_. Moreover, Ag_3_PO_4_ is slightly soluble in aqueous solution, which could also decrease its structural stability during the photocatalytic process. To further improve the photocatalytic performance as well as the photocatalytic stability of Ag_3_PO_4_, much recent work has been devoted to the heterojunction composites constructed from Ag_3_PO_4_ and other semiconductors, such as AgX/Ag_3_PO_4_ (*X* = Cl, Br, I), Fe_3_O_4_/Ag_3_PO_4_, Ag_3_PO_4_/SnO_2_, Ag_3_PO_4_/TiO_2_, Ag_3_PO_4_/Bi_2_MoO_6_, g-C_3_N_4_/Ag_3_PO_4_, Ag_3_PO_4_/CeO_2_, Ag_3_PO_4_/SrTiO_3_, Ag_3_PO_4_/BiPO_4_, Ag_3_PO_4_/MoS_2_, and Ag_3_PO_4_/g-C_3_N_4_ [33–46]. In these composite photocatalysts, photogenerated electrons and holes tend to migrate from one semiconductor to another, leading to an efficient separation of electron hole pairs. As a result, more photogenerated electrons and/or holes are available for participating in photocatalytic reactions. It has been shown that the heterojunction composites exhibit enhanced photocatalytic performance compared to individual semiconductors. Furthermore, the photocorrosion and solubility behaviors of Ag_3_PO_4_ can be inhibited to some extent by the construction of heterojunction composites.

In this work, we report the integration of Ag_3_PO_4_ with Bi_4_Ti_3_O_12_ to form Ag_3_PO_4_/Bi_4_Ti_3_O_12_ heterojunction composites. Bi_4_Ti_3_O_12_ has a layered structure composed of alternate (Bi_2_Ti_3_O_10_)^2−^ blocks and (Bi_2_O_2_)^2+^ layers along the *c*-axis orientation [47]. Density functional theory calculation has shown that the conduction band (CB) and valence band (VB) of Bi_4_Ti_3_O_12_ consist of Ti 3d + Bi 6p orbitals and O 2p + Bi 6 s hybrid orbitals, respectively [48]. Due to its unique layered crystal structure and electronic band structure, Bi_4_Ti_3_O_12_ exhibits pronounced photocatalytic activity toward the degradation of organic pollutants [49–53]. It is known that Ag_3_PO_4_ is a p-type semiconductor and Bi_4_Ti_3_O_12_ is an n-type semiconductor. The well-matched overlapping band-structures suggest that Ag_3_PO_4_ and Bi_4_Ti_3_O_12_ can be used to construct an excellent p-n heterojunction composite photocatalyst with super photocatalytic performance.

## Methods

### Synthesis of Bi_4_Ti_3_O_12_ and Ag_3_PO_4_ nanoparticles

Bi_4_Ti_3_O_12_ nanoparticles were synthesized via a polyacrylamide gel route as described in the literature [54]. All raw materials and chemical reagents were of analytical grade and were used without further purification. Five milligrams of HNO_3_ was added to 20 mL distilled water to make a dilute nitric acid solution. 0.00857 mol of Bi(NO_3_)_3_·5H_2_O was dissolved in the above dilute nitric acid solution (designated as solution A). 0.00643 mol of C_16_H_36_O_4_Ti was dissolved in 25 mL ethanol (designated as solution B). The solution B was added slowly into the solution A to obtain a mixture solution. Then, the mixture solution were successively added with 0.0225 mol of citric acid, 20 g of glucose, and 0.135 mol of acrylamide. During the addition of chemical reagents, the solution was agitated by magnetic stirring to make the additives dissolve fully. The resultant solution was heated in a water bath at 80 °C to initiate the polymerization reaction. After dried at 120 °C for 24 h in a thermostat drier, the formed xerogel was ground into powder and submitted to calcination in a tubular furnace at 300 °C for 3 h and then at 500 °C for 8 h. After the tubular furnace was naturally cooled down to room temperature, Bi_4_Ti_3_O_12_ nanoparticles were obtained.

Ag_3_PO_4_ nanoparticles were synthesized by an ion exchange method. 0.003 mol of AgNO_3_ and 0.001 mol of Na_2_HPO_4_ were dissolved in 30 and 20 mL distilled water with the aid of magnetic stirring, respectively. The Na_2_HPO_4_ solution was added drop by drop to the AgNO_3_ solution under continuous stirring. The mixture solution was then continuously stirred by a magnetic stirrer for 5 h, during which time Ag_3_PO_4_ nanoparticles were formed. The produced particles were collected and washed several times with distilled water and absolute ethanol, followed by drying at 60 °C for 10 h.

### Preparation of Bi_4_Ti_3_O_12_/Ag_3_PO_4_ nanocomposites

A stoichiometric amount of the as-prepared Bi_4_Ti_3_O_12_ nanoparticles was added to 30 mL distilled water and was submitted to ultrasonic treatment for 1 h to make the particles disperse uniformly. To the suspension was dissolved 0.003 mol of AgNO_3_. 0.001 mol of Na_2_HPO_4_ was dissolved in 20 mL distilled water, which was then added drop by drop to the above suspension. The resultant mixture solution was magnetically stirred for 5 h, during which time Ag_3_PO_4_ nanoparticles were grown and integrated with Bi_4_Ti_3_O_12_ nanoparticles to form Bi_4_Ti_3_O_12_/Ag_3_PO_4_ composites. The produced composites were collected, washed several times with distilled water and absolute ethanol, and dried at 60 °C for 10 h. By varying the Bi_4_Ti_3_O_12_ content from 5 to 15%, several composite samples of 5% Bi_4_Ti_3_O_12_/Ag_3_PO_4_, 10% Bi_4_Ti_3_O_12_/Ag_3_PO_4_, and 15% Bi_4_Ti_3_O_12_/Ag_3_PO_4_ were prepared.

### Sample characterization

X-ray powder diffraction (XRD) with Cu Kα radiation was used to determine the crystal structure of the samples. Field emission scanning electron microscopy (SEM) and field emission transmission electron microscopy (TEM) were used to investigate the morphology and microstructure of the samples. The optical absorption and bandgap energy of the samples was investigated by ultraviolet-visible diffuse reflectance spectroscopy (UV-vis DRS) on a UV-vis spectrophotometer with an integrating sphere attachment. The chemical composition and electron binding energies for the elements were measured by X-ray photoelectron spectroscopy (XPS) on a PHI-5702 multifunctional X-ray photoelectron spectrometer, where the binding energy scale of the XPS data was calibrated against the adventitious C 1s peak at the binding energy of 284.8 eV. The photoluminescence (PL) spectrum of the samples was measured by using a fluorescence spectrophotometer (excitation wavelength 315 nm).

### Photoelectrochemical measurement

The photoelectrochemical properties of the photocatalysts were measured by electrochemical impedance spectroscopy (EIS) and photocurrent response on a CST 350 electrochemical workstation using a three-electrode cell configuration [55]. A platinum foil was used as the counter electrode, and a standard calomel electrode (SCE) was used as the reference electrode. The working electrode was prepared as follows: 15 mg of the photocatalysts and 0.75 mg of polyvinylidene fluoride (PVDF) were mixed together using 1-methyl-2-pyrrolidione (NMP) as solvent to form slurry. The slurry was uniformly coated onto fluorine-doped tin oxide (FTO) glass substrate with an area of 1 cm × 1 cm and then submitted to drying at 60 °C for 5 h in a thermostat drying oven. Na_2_SO_4_ aqueous solution with concentration of 0.1 mol L^−1^ was used as the electrolyte. The EIS measurement was carried out by the use of the sinusoidal voltage pulse with amplitude of 5 mV and in the frequency range of 10^−2^– 10^5^ Hz. The transient photocurrent response was measured at a bias potential of 0.2 V. During the photoelectrochemical measurements, the working electrode was irradiated by a 200 W xenon lamp.

### Photocatalytic evaluation

Rhodamine B (RhB) was chosen as the target organic pollutant to evaluate its degradation behavior over the samples under irradiation from a 200 W xenon lamp (solar simulator). RhB was dissolved in distilled water to make 5 mg L^−1^ RhB aqueous solution. Twenty milligrams of the photocatalyst was loaded in 100 mL of RhB solution. The mixed suspension was firstly stirred by a magnetic stirrer for 20 min in the dark and then submitted to photocatalysis. During the photocatalysis process, the reactor was cooled with a water cooling system to maintain the reaction solution at room temperature. At given time intervals, a small portion of the reaction solution was taken out from the reactor for examining the RhB concentration, which was determined by measuring the absorbance of the solution at λ = 554 nm on a UV-vis spectrophotometer. Before absorbance measurement, the photocatalyst was removed by centrifugalization. The percentage degradation of RhB is defined as (*C*_0_ − *C*_*t*_)/*C*_0_ × 100%, where *C*_0_ is the initial RhB concentration and *C*_*t*_ is the remaining RhB concentration after photocatalysis for time *t*.

### Detection of hydroxyl

PL spectroscopy is an important technique that can be used to detect hydroxyl (•OH) radicals formed over the simulated sunlight irradiated photocatalyst. Here, terephthalic acid (TPA) was used as the •OH scavenger to examine •OH radicals. NaOH solution with concentration of 1.0 mmol L^−1^ was prepared by dissolving NaOH in distilled water. A stoichiometric amount of TPA was dissolved in the above NaOH solution to make 0.25 mmol L^−1^ TPA solution. Twenty milligrams of the photocatalyst was loaded in 100 mL of the TPA solution. The mixture was magnetically stirred for 20 min in the dark and then irradiated by a 200-W xenon lamp. A small portion of the solution was taken out from the reactor after reaction for a certain period of time and submitted to centrifugation to remove the photocatalyst. The PL spectrum of the clear solution was measured on a fluorescence spectrophotometer (excitation wavelength 315 nm).

## Results and discussion

### XRD analysis

**Fig. 1:**
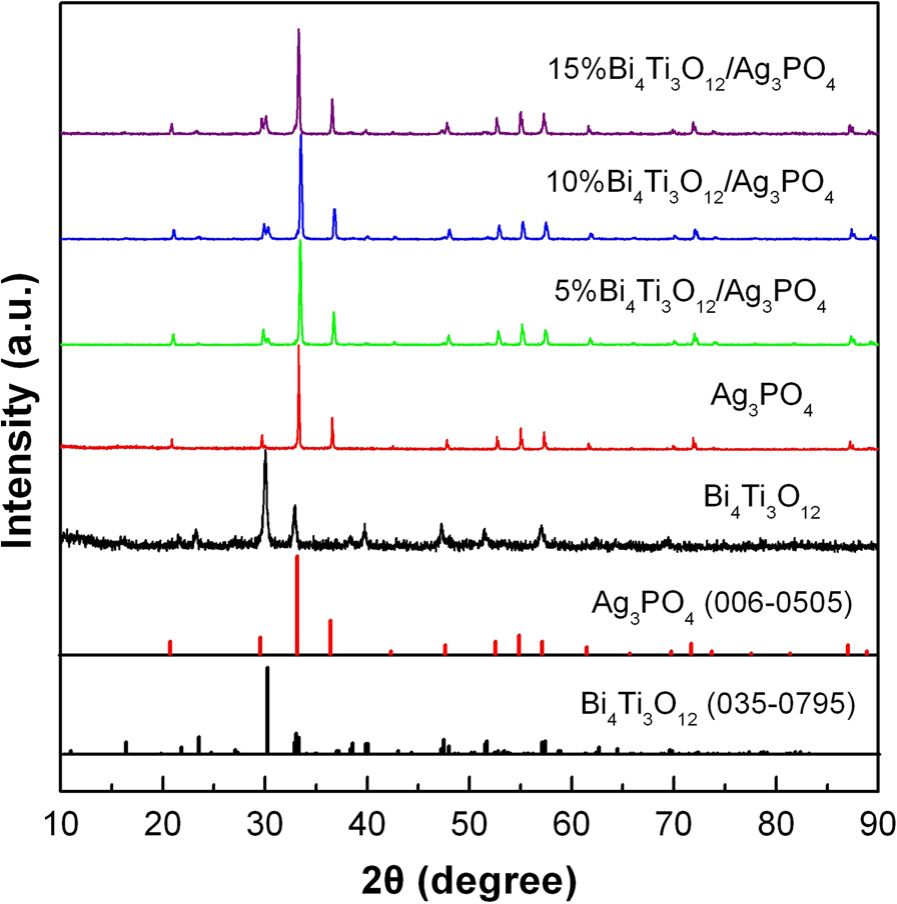
XRD patterns of Bi_4_Ti_3_O_12_, Ag_3_PO_4_, and Bi_4_Ti_3_O_12_/Ag_3_PO_4_ composites

Figure 1 shows the XRD patterns of Bi_4_Ti_3_O_12_, Ag_3_PO_4_, 5% Bi_4_Ti_3_O_12_/Ag_3_PO_4_, 10% Bi_4_Ti_3_O_12_/Ag_3_PO_4_, and 15% Bi_4_Ti_3_O_12_/Ag_3_PO_4_ samples. The standard XRD line patterns for Bi_4_Ti_3_O_12_ orthorhombic structure (JCPDS card no. 035-0795) and Ag_3_PO_4_ cubic structure (JCPDS card no. 006-0505) are also shown in Fig. 1. For Bi_4_Ti_3_O_12_ sample, all diffraction peaks are in good agreement with those in the JCPDS card no. 035-0795, indicating that the sample crystallizes in a pure Bi_4_Ti_3_O_12_ orthorhombic phase [54]. For Ag_3_PO_4_ sample, all diffraction peaks can be indexed according to the standard diffraction lines in the JCPDS card no. 006-0505, implying the formation of pure Ag_3_PO_4_ cubic phase [31]. For the composites, the XRD patterns can be indexed into two sets of diffraction peaks corresponding to Bi_4_Ti_3_O_12_ and Ag_3_PO_4_ phases. The peak intensity from Bi_4_Ti_3_O_12_ phase increases with increasing its content, which is clearly seen from the Bi_4_Ti_3_O_12_ (171) diffraction peak at 30.1^o^. No diffraction peaks assignable to other impurity phases are detected, indicating that Bi_4_Ti_3_O_12_ and Ag_3_PO_4_ undergo no structural change in the composites.

### SEM analysis

**Fig. 2:**
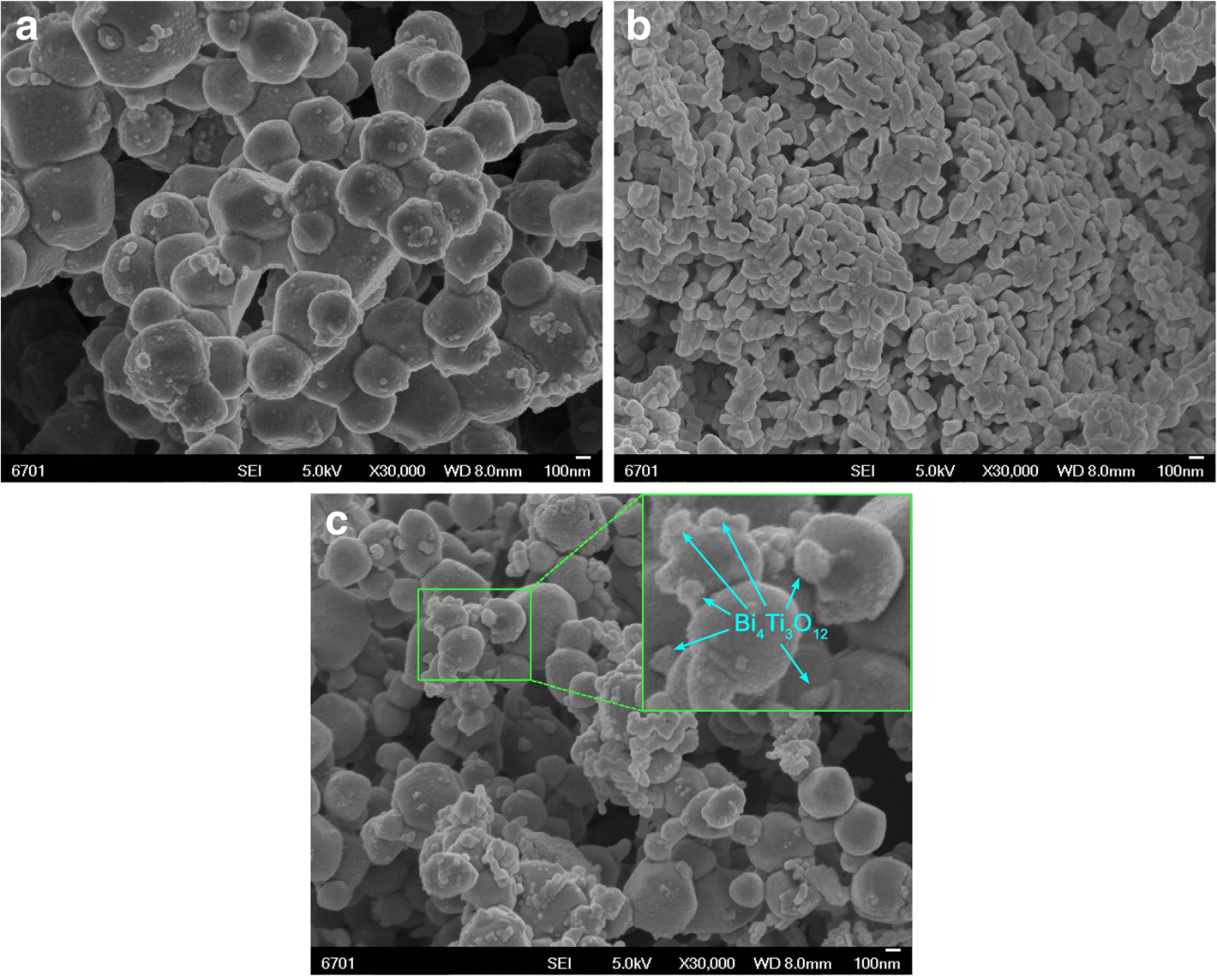
SEM images of **a** Ag_3_PO_4_, **b** Bi_4_Ti_3_O_12_, and **c** 10%Bi_4_Ti_3_O_12_/Ag_3_PO_4_

Figure 2a–c shows the SEM images of Ag_3_PO_4_, Bi_4_Ti_3_O_12_, and 10% Bi_4_Ti_3_O_12_/Ag_3_PO_4_ samples, respectively. The SEM image given in Fig. 2a shows that the Ag_3_PO_4_ particles have sphere-like morphology with size ranging from 300 to 600 nm. The SEM image shown in Fig. 2b reveals that the Bi_4_Ti_3_O_12_ particles present sphere-like or ellipsoid-like morphology and have a size distribution range of 60–90 nm. From the SEM image in Fig. 2c, one can see that small-sized Bi_4_Ti_3_O_12_ particles are assembled onto the surface of large-sized Ag_3_PO_4_ particles to form Bi_4_Ti_3_O_12_/Ag_3_PO_4_ heterostructure, as indicated by arrows.

### TEM analysis

**Fig. 3:**
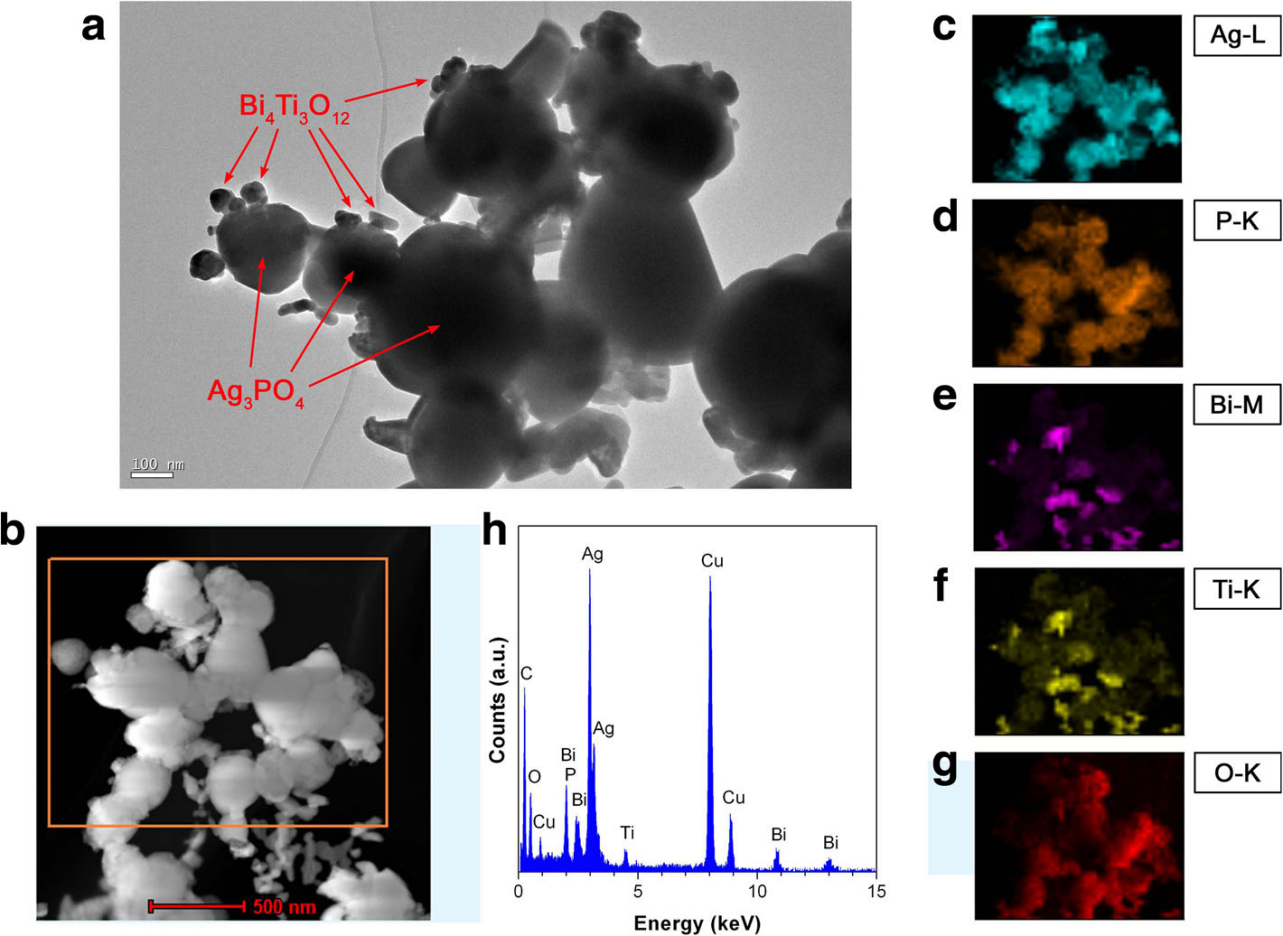
**a** TEM image of 10% Bi_4_Ti_3_O_12_/Ag_3_PO_4_. **b** DF-STEM image of 10% Bi_4_Ti_3_O_12_/Ag_3_PO_4_. **c**–**g** The corresponding elemental mapping images of the region indicated in **b**. **h** EDS spectrum of 10% Bi_4_Ti_3_O_12_/Ag_3_PO_4_

TEM was used to further investigate the microstructure of Bi_4_Ti_3_O_12_/Ag_3_PO_4_ composites. Figure 3a shows the TEM image of 10% Bi_4_Ti_3_O_12_/Ag_3_PO_4_. The large-sized particles are identified to be Ag_3_PO_4_ particles, which have a spherical morphology with size of several hundred nanometers. Much smaller-sized particles with diameter of several tens of nanometers, which are identified to be Bi_4_Ti_3_O_12_ nanoparticles, are seen to be assembled onto Ag_3_PO_4_ particles, as indicated by arrows. The TEM observation indicates the formation of Bi_4_Ti_3_O_12_/Ag_3_PO_4_ heterostructure, which agrees with that observed from the SEM image. Figure 3b shows the dark field scanning TEM (DF-STEM) image of 10% Bi_4_Ti_3_O_12_/Ag_3_PO_4_. The corresponding elemental mapping images of the region indicated in Fig. 3b are given in Fig. 3c–g. It is seen that the large-sized particles present the elemental distribution of Ag and P and are therefore confirmed to be Ag_3_PO_4_ particles. The small-sized particles anchored onto Ag_3_PO_4_ particles display the elemental distribution of Bi and Ti, confirming that they are Bi_4_Ti_3_O_12_ particles. The elemental mapping images further reveal the integration of Bi_4_Ti_3_O_12_ with Ag_3_PO_4_ to form Bi_4_Ti_3_O_12_/Ag_3_PO_4_ heterostructure. Furthermore, the elements Ag and P, as well as Bi and Ti, have an identical distribution, implying no chemical composition segregation in the Ag_3_PO_4_ and Bi_4_Ti_3_O_12_ phases. The O element distribution is observed through the whole composites. Energy-dispersive X-ray spectroscopy (EDS) was further used to analyze the chemical composition of 10% Bi_4_Ti_3_O_12_/Ag_3_PO_4_. As shown in Fig. 3h, the signals of the constituent elements of the composite are clearly included in the spectrum. The observed C and Cu signals could derive from the microgrid that is used for supporting the sample. It is noted that EDS is suitably used for the quantitative determination of the content of heavy elements (e.g., Bi, Ti, and Ag), but not light elements (e.g., P and O) [56]. The atomic ratio of Bi to Ti is obtained as 4/3 from the EDS spectrum, which agrees well the Bi/Ti atomic ratio of Bi_4_Ti_3_O_12_ phase. The atomic ratio of Ti/Ag is very close to 1/9, implying that Bi_4_Ti_3_O_12_ phase accounts for about 10% of the total molar content of the composite.

### BET surface analysis

**Fig. 4:**
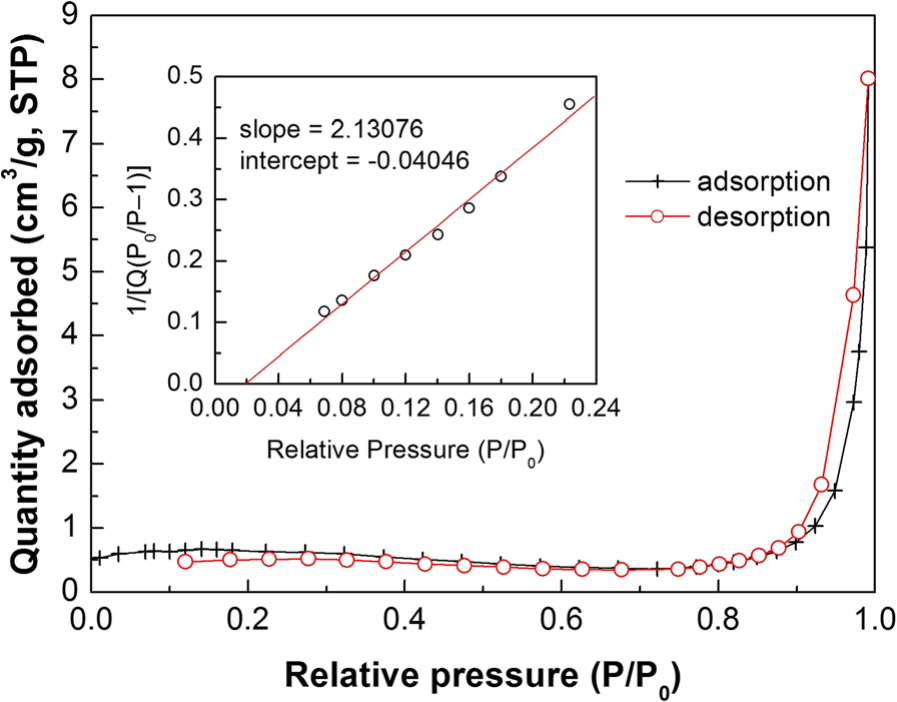
N_2_ adsorption-desorption isotherm and BET surface area plot of 10% Bi_4_Ti_3_O_12_/Ag_3_PO_4_

Figure 4 shows the N_2_ adsorption-desorption isotherm of 10% Bi_4_Ti_3_O_12_/Ag_3_PO_4_. This kind of isotherm is very similar to type II adsorption isotherm according to the IUPAC classification. The desorption curve coincides almost with the adsorption curve with no obvious hysteresis loop, implying the absence of mesopores in the composite. The insert in Fig. 4 shows the Brunauer-Emmett-Teller (BET) surface area plot of the composite, from which the BET surface area is calculated to be 2.08 m^2^ g^−1^.

### XPS analysis

**Fig. 5:**
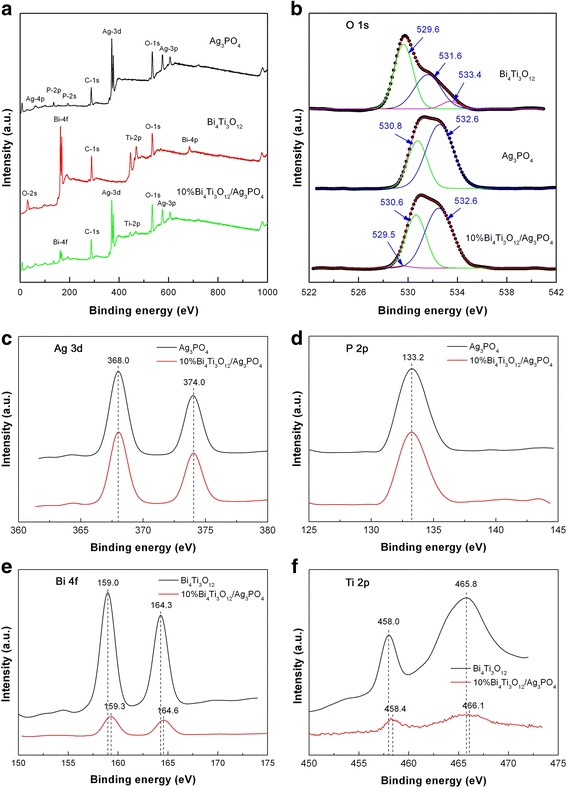
XPS spectra of Bi_4_Ti_3_O_12_, Ag_3_PO_4_, and 10% Bi_4_Ti_3_O_12_/Ag_3_PO_4_. **a** XPS survey scan spectra. **b** O 1s XPS spectra. **c** Ag 3d XPS spectra. **d** P 2p XPS spectra. **e** Bi 4f XPS spectra. **f** Ti 2p XPS spectra

The chemical composition and elemental oxidation state of Ag_3_PO_4_, Bi_4_Ti_3_O_12_, and 10% Bi_4_Ti_3_O_12_/Ag_3_PO_4_ were also investigated by XPS. Figure 5a shows the XPS survey scan spectra of the samples, revealing that the samples clearly include their own constituent elements and no other impurity elements are found. Figure 5b shows the O 1s XPS spectra of the samples. For bare Bi_4_Ti_3_O_12_, the O 1s XPS signal can be fitted into three peaks at 529.6, 531.6, and 533.4 eV. The binding energy at 529.6 eV is attributed to the contribution of the crystal lattice oxygen. The peaks at higher binding energies of 531.6 and 533.4 eV could arise due to surface defects and chemisorbed oxygen species [55]. For bare Ag_3_PO_4_, the O 1s XPS spectrum is deconvoluted into two peaks at 530.8 and 532.6 eV, which are ascribed to the crystal lattice oxygen and the surface adsorbed oxygen, respectively. For 10% Bi_4_Ti_3_O_12_/Ag_3_PO_4_, the O 1s binding energy of the crystal lattice oxygen in Bi_4_Ti_3_O_12_ and Ag_3_PO_4_ is observed at 529.5 and 530.6 eV, respectively. A slight downshift of the O 1s binding energy peaks is observed in the composite, implying the possible chemical bonding between Bi_4_Ti_3_O_12_ and Ag_3_PO_4_. Figure 5c, d shows the XPS spectra of Ag 3d and P 2p, respectively. The peaks at 368.0 and 374.0 eV belong to the binding energies for Ag 3d_5/2_ and Ag 3d_3/2_, respectively, suggesting the presence of Ag^+^ oxidation state. The presence of Ag^0^ metal state in the samples can be excluded because no additional peaks are visible on the Ag 3d XPS spectra [37]. The observation of the P 2p binding energy at 133.2 eV is indicative of the presence of P^5+^ oxidation state [37]. The Ag 3d and P 2p XPS signals undergo no change in the composite. Figure 5e, f shows the XPS spectra of Bi 4f and Ti 2p, respectively. For bare Bi_4_Ti_3_O_12_, the Bi 4f spectrum presents two sharp peaks at 159.0 and 164.3 eV, which correspond to the binding energies of Bi 4f_7/2_ and Bi 4f_5/2_, respectively [52]. This implies that Bi exists in the + 3 oxidation state. On the Ti 2p spectrum, the peaks at 458.0 and 465.8 eV are assigned to the binding energies for Ti 2p_3/2_ and Ti 2p_1/2_, respectively, which indicates that Ti is in the form of Ti^4+^ oxidation state [52]. For the composite, the Bi 4f and Ti 2p peaks exhibit a slight shift toward higher binding energies, which could arise due to the chemical bonding between Bi_4_Ti_3_O_12_ and Ag_3_PO_4_.

### UV-vis DRS spectra

**Fig. 6:**
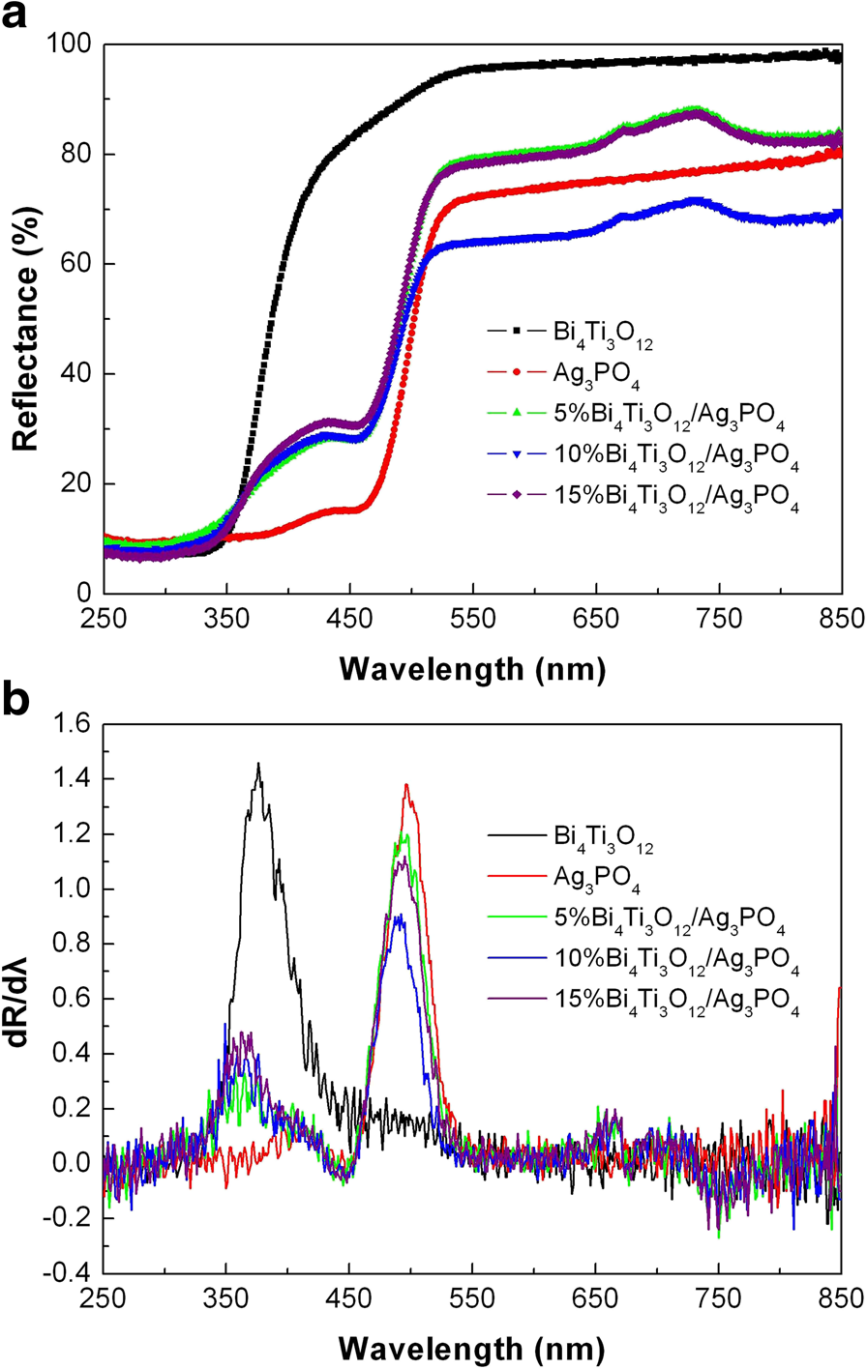
**a** UV-vis DRS spectra of Bi_4_Ti_3_O_12_, Ag_3_PO_4_, and Bi_4_Ti_3_O_12_/Ag_3_PO_4_ composites. **b** The corresponding first derivative spectra

Figure 6a shows the UV-vis DRS spectra of Bi_4_Ti_3_O_12_, Ag_3_PO_4_, and Bi_4_Ti_3_O_12_/Ag_3_PO_4_ composites, and Fig. 6b gives the corresponding first derivative of the spectra. The absorption edge of the samples can be derived from the peaks on the first derivative spectra. It is seen that Bi_4_Ti_3_O_12_ has an absorption edge at 376.9 nm, and Ag_3_PO_4_ has an absorption edge at 498.5 nm. The absorption edges arise due to the electron transition from the valence band to the conduction band of the semiconductors, from which the bandgap energies (*E*_g_) of Bi_4_Ti_3_O_12_ and Ag_3_PO_4_ are obtained to be 3.29 and 2.49 eV, respectively. For the composites, the absorption edges of Bi_4_Ti_3_O_12_ and Ag_3_PO_4_ exhibit a slight shift toward the short wavelength, which could be due to their mutual effect of light absorption on each other. The bandgap energies of Bi_4_Ti_3_O_12_ and Ag_3_PO_4_ are expected to undergo a negligible change in the composites.

### PL spectra

**Fig. 7:**
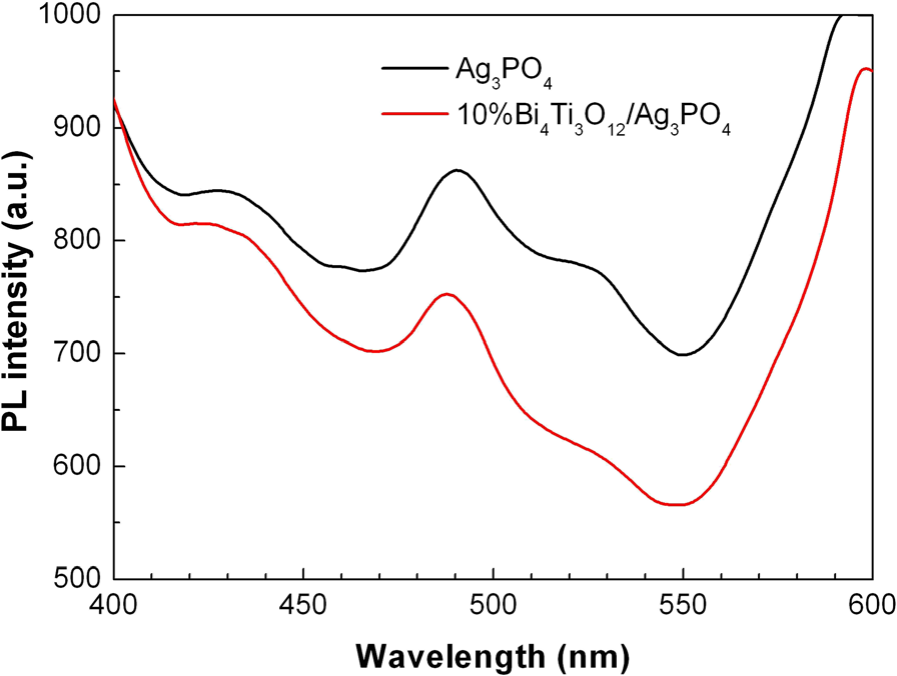
PL spectra of Ag_3_PO_4_ and 10% Bi_4_Ti_3_O_12_/Ag_3_PO_4_

PL spectroscopy is used to evaluate the recombination behavior of photogenerated electron-hole pairs in the photocatalysts. The PL intensity is in proportion to the recombination rate of photogenerated electrons and holes. Figure 7 shows the PL spectra of Ag_3_PO_4_ and 10% Bi_4_Ti_3_O_12_/Ag_3_PO_4_ measured at an excitation wavelength of 315 nm. For Ag_3_PO_4_ particles, three PL emission peaks are observed at around 430, 490, and 525 nm. In contrast, the intensity of the PL emission peaks from 10% Bi_4_Ti_3_O_12_/Ag_3_PO_4_ composite is clearly decreased, implying a decrease in the electron-hole recombination. The efficient separation of electron-hole pairs is attributed to the charge transfer between Bi_4_Ti_3_O_12_ and Ag_3_PO_4_. As a result, photogenerated electrons and holes in Bi_4_Ti_3_O_12_/Ag_3_PO_4_ composites are increasingly available for the photocatalytic reactions.

### Photoelectrochemical properties

**Fig. 8:**
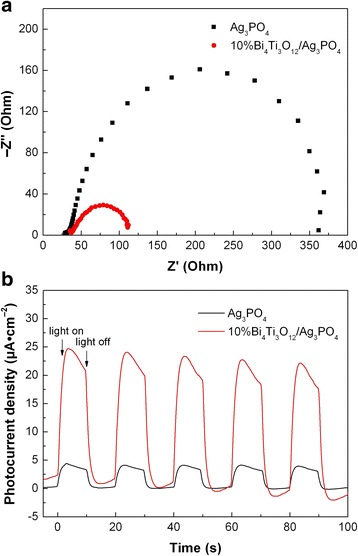
**a** Nyquist plots of the EIS spectra for Ag_3_PO_4_ and 10% Bi_4_Ti_3_O_12_/Ag_3_PO_4_. **b** Transient photocurrent responses of Ag_3_PO_4_ and 10% Bi_4_Ti_3_O_12_/Ag_3_PO_4_

EIS and photocurrent response can be also used to investigate the separation and transfer behavior of photogenerated electrons and holes in the photocatalysts. Figure 8a shows the Nyquist plots of the EIS spectra for Ag_3_PO_4_ and 10% Bi_4_Ti_3_O_12_/Ag_3_PO_4_ electrodes, which present a typical semicircle. The diameter of the semicircle is related to the charge-transfer resistance at the electrode/electrolyte interface. It is obvious that the Nyquist plot of 10% Bi_4_Ti_3_O_12_/Ag_3_PO_4_ shows a much smaller semicircle diameter than that of bare Ag_3_PO_4_, indicating that the composite has a relatively smaller charge-transfer resistance under simulated sunlight irradiation. The observation of smaller charge-transfer resistance implies an increased separation efficiency of photogenerated electron-hole pairs and fast interface charge transfer occurring in the 10% Bi_4_Ti_3_O_12_/Ag_3_PO_4_ composite. Figure 8b shows the transient photocurrent responses of Ag_3_PO_4_ and 10%Bi_4_Ti_3_O_12_/Ag_3_PO_4_ recorded for several switch-on and switch-off cycles under intermittent irradiation of simulated sunlight. It is seen that the transient photocurrent responses are highly reproducible when the light is repeatedly switched between on and off. During the irradiation period, the photocurrent density of 10% Bi_4_Ti_3_O_12_/Ag_3_PO_4_ is obtained as ~ 24 μA cm^−2^, which is much higher than that of bare Ag_3_PO_4_ (~ 4 μA cm^−2^), indicating a more efficient separation of photogenerated electron-hole pairs in the composite due to the carrier transfer between Bi_4_Ti_3_O_12_ and Ag_3_PO_4_.

### Photocatalytic performances

**Fig. 9:**
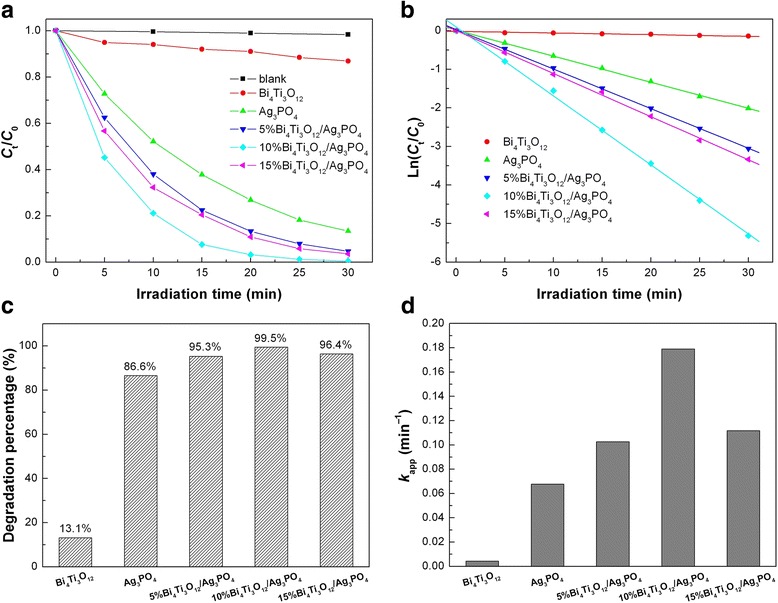
**a** Time-dependent photocatalytic degradation of RhB over Bi_4_Ti_3_O_12_, Ag_3_PO_4_, and Bi_4_Ti_3_O_12_/Ag_3_PO_4_ composites. **b** Plots of Ln(*C*_*t*_/*C*_0_) versus irradiation time *t* for the samples. **c** Degradation percentages of RhB after 30 min of photocatalysis over the samples. **d** Apparent first-order reaction rate constants *k*_app_ for the samples

Figure 9a shows the time-dependent photocatalytic degradation of RhB over Bi_4_Ti_3_O_12_, Ag_3_PO_4_, and Bi_4_Ti_3_O_12_/Ag_3_PO_4_ composites under simulated sunlight irradiation, along with the blank experiment result. Without loading the photocatalyst, RhB exhibits a good stability under simulated sunlight irradiation and its percentage degradation is only about 1.7% after 30 min of irradiation. When Ag_3_PO_4_ is used as the photocatalyst, RhB undergoes a substantial degradation with increasing the irradiation time, while a relatively weak degradation of the dye is observed for Bi_4_Ti_3_O_12_. More importantly, when Ag_3_PO_4_ is integrated with Bi_4_Ti_3_O_12_, the formed Bi_4_Ti_3_O_12_/Ag_3_PO_4_ heterojunction composites exhibit significantly enhanced photocatalytic activity compared to bare Ag_3_PO_4_ or Bi_4_Ti_3_O_12_. The highest photocatalytic activity is observed for 10% Bi_4_Ti_3_O_12_/Ag_3_PO_4_, where the percentage degradation of RhB after 30 min of photocatalysis reaches 99.5%, as shown in Fig. 9c. To further reveal the photocatalytic activity of the samples, the photocatalytic degradation kinetics of RhB is investigated. Figure 9b shows the plots of Ln(*C*_*t*_/*C*_0_) versus irradiation time (*t*) for the samples. It is seen that the dye degradation conforms well to the first-order kinetic equation Ln(*C*_*t*_/*C*_0_) = − *k*_app_ *t*, where *k*_app_ is the apparent first-order reaction rate constant (min^−1^) [57]. The obtained rate constants for the samples are shown in Fig. 9d. The rate constant *k*_app_ for 10% Bi_4_Ti_3_O_12_/Ag_3_PO_4_ is obtained as 0.17891 min^−1^, compared to 0.06764 min^−1^ for Ag_3_PO_4_. This implies that the photocatalytic activity of 10% Bi_4_Ti_3_O_12_/Ag_3_PO_4_ is about 2.6 times higher than that of bare Ag_3_PO_4_.

**Fig. 10:**
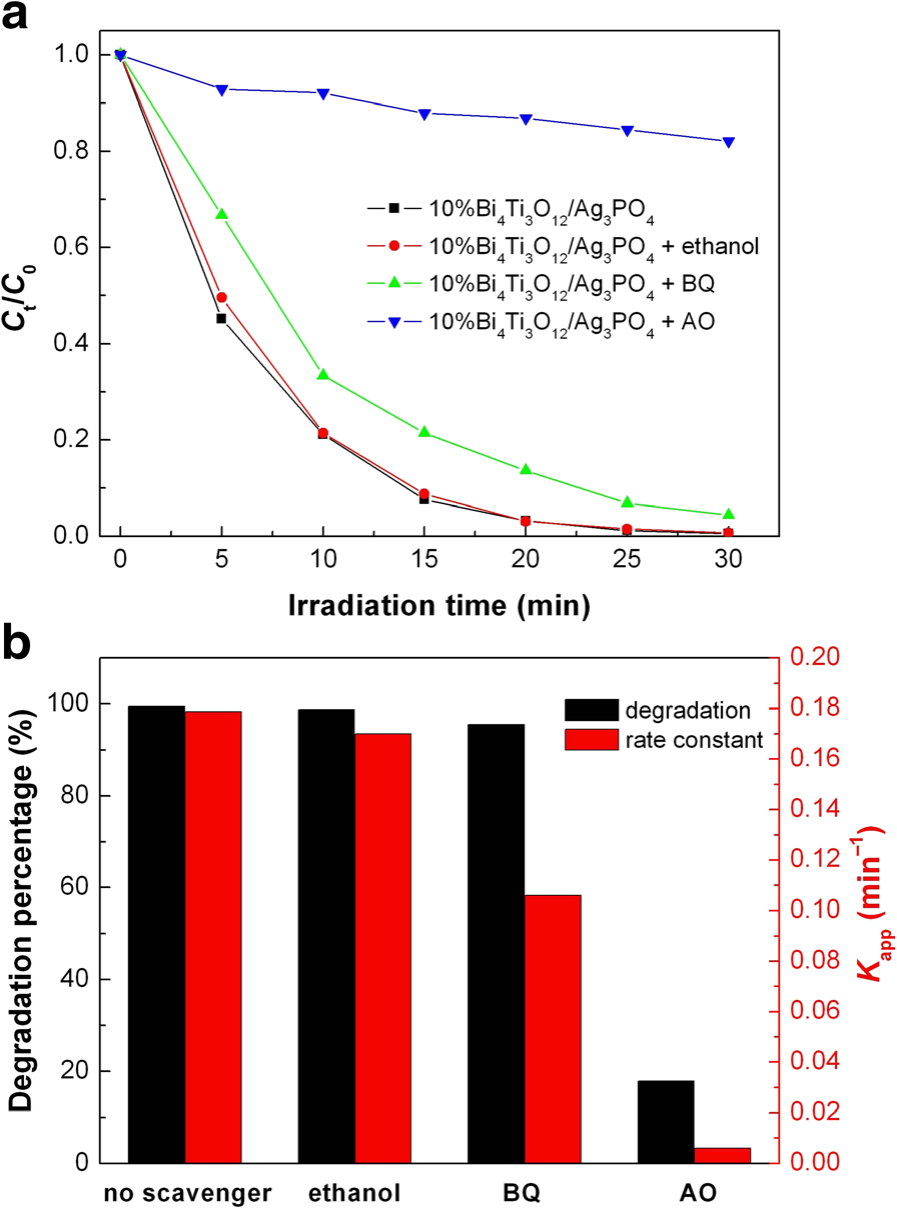
Effect of ethanol, BQ, and AO on the RhB degradation over 10% Bi_4_Ti_3_O_12_/Ag_3_PO_4_. **a** Time-dependent photocatalytic degradation of RhB. **b** Degradation percentages of RhB after 30 min of photocatalysis and first-order reaction rate constants *k*_app_

In most of the photocatalytic systems, the dominant active species responsible for the dye degradation include •OH, superoxide (•O_2_^−^), and h^+^ [58]. It is known that •OH, •O_2_^−^, and h^+^ can be scavenged by ethanol, benzoquinone (BQ), and ammonium oxalate (AO), respectively [56]. Therefore, the role of the active species in the photocatalysis can be verified by investigating the effect of ethanol, BQ, and AO on the photocatalytic degradation of the dye. Five milligrams of ethanol, 0.0011 g of BQ, and 0.0142 g of AO are separately added in 100 mL reaction solution, and then, the photocatalytic experiments are carried out under the same conditions. Figure 10a shows the effect of the scavengers on the degradation of RhB over 10% Bi_4_Ti_3_O_12_/Ag_3_PO_4_. The degradation percentages of RhB after 30 min of photocatalysis and the rate constants are shown in Fig. 10b. It is found that the addition of ethanol to the reaction solution has a negligible effect on the degradation of RhB, implying that •OH plays little or no role in the dye degradation. However, the addition of BQ or AO leads to an obvious suppression on the dye degradation. Particularly, only 17.9% of RhB is observed to be degraded with the addition of AO. This suggests that h^+^ and •O_2_^−^ are the dominant and secondary reactive species causing the dye degradation, respectively.

### Information of •OH radicals

**Fig. 11:**
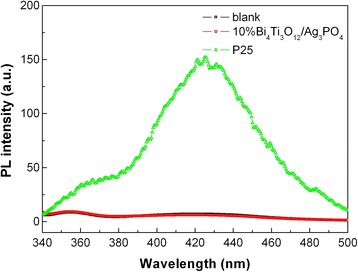
PL spectra of the TPA solution reacted for 30 min over 10% Bi_4_Ti_3_O_12_/Ag_3_PO_4_ and P25

We further examine whether there are •OH radicals formed over the simulated sunlight-irradiated 10% Bi_4_Ti_3_O_12_/Ag_3_PO_4_ by PL spectroscopy using TPA as the scavenger of •OH. It is known that TPA will react with •OH to produce 2-hydroxyterephthalic acid (TAOH) that can emit photoluminescence having a wavelength of 429 nm [59]. The PL emission intensity is proportional to the amount of •OH radicals. Figure 11 shows the PL spectra of the TPA solution after reaction for 30 min over 10% Bi_4_Ti_3_O_12_/Ag_3_PO_4_ and P25. It is well established that •OH is easily generated over the irradiated P25 (a mixed-phase TiO_2_ photocatalyst) in water solution. As a result, the TPA reaction solution shows a strong PL signal at 429 nm when P25 is used as the photocatalyst. However, when 10% Bi_4_Ti_3_O_12_/Ag_3_PO_4_ is used as the photocatalyst, the TPA reaction solution is very similar to the blank TPA solution, showing no obvious PL signal at 429 nm. This indicates that no •OH radicals are produced in the 10% Bi_4_Ti_3_O_12_/Ag_3_PO_4_ photocatalytic system.

### Discussion of photocatalytic mechanism

**Fig. 12:**
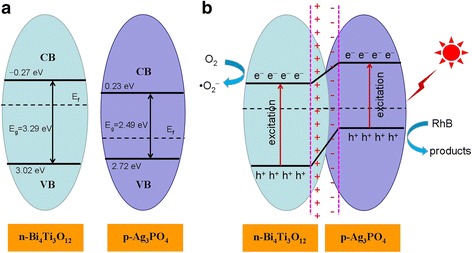
Schematic illustration of the energy band structures for **a** separate Bi_4_Ti_3_O_12_ and Ag_3_PO_4_ and **b** Bi_4_Ti_3_O_12_/Ag_3_PO_4_ heterojunction

The CB and VB edge potentials of Bi_4_Ti_3_O_12_ and Ag_3_PO_4_ are determined according the method described in the literature [60], as schematically shown in Fig. 12a. It is seen that the CB potential of Bi_4_Ti_3_O_12_ is negative to that of Ag_3_PO_4_, and moreover, Bi_4_Ti_3_O_12_ is intrinsically an n-type semiconductor and Ag_3_PO_4_ behaves as a p-type semiconductor. This indicates that when the two semiconductors are integrated to form p-n heterojunction, electrons will migrate from Bi_4_Ti_3_O_12_ to Ag_3_PO_4_, leaving behind positive charge centers at the interface of Bi_4_Ti_3_O_12_ and negative charge centers at the interface of Ag_3_PO_4_. Simultaneously, an internal electric field is created at the interface region of the Bi_4_Ti_3_O_12_/Ag_3_PO_4_ heterojunction, as shown in Fig. 12b. The direction of the internal electric field is from Bi_4_Ti_3_O_12_ to Ag_3_PO_4_. Under the action of the internal electric field, photogenerated electrons will migrate from Ag_3_PO_4_ to Bi_4_Ti_3_O_12_ and conversely the photogenerated holes will migrate from Bi_4_Ti_3_O_12_ to Ag_3_PO_4_. Due to the carrier transfer process, the recombination of electron-hole pairs can be effectively inhibited. As a result, more photogenerated holes and electrons are able to participate in the photocatalytic reactions, thus leading to an increased photocatalytic performance of the Bi_4_Ti_3_O_12_/Ag_3_PO_4_ heterojunction composites. It is noted that the photocatalytic performance of the composite photocatalysts is highly associated with the number of formed heterostructures. Generally, a proper proportion between two semiconductors is required for the creation of a large number of heterostructures in the composites. In the Bi_4_Ti_3_O_12_/Ag_3_PO_4_ composites, the optimum content of Bi_4_Ti_3_O_12_ is about 10%, and at this Bi_4_Ti_3_O_12_ content, the formed composite (i.e., 10%Bi_4_Ti_3_O_12_/Ag_3_PO_4_) exhibits the highest photocatalytic activity.

## Conclusions

Bi_4_Ti_3_O_12_/Ag_3_PO_4_ heterojunction composites were prepared by an ion-exchange method. Compared to bare Bi_4_Ti_3_O_12_ and Ag_3_PO_4_ particles, the as-prepared Bi_4_Ti_3_O_12_/Ag_3_PO_4_ composites exhibit an enhanced photocatalytic activity toward the degradation of RhB under simulated sunlight irradiation. The highest photocatalytic activity is observed for the composite with Bi_4_Ti_3_O_12_ fraction of 10%, which is about 2.6 times higher than that of bare Ag_3_PO_4_. The enhanced photocatalytic activity of the composites can be explained by the efficient separation of photogenerated electron-hole pairs due to the migration of the carriers between Bi_4_Ti_3_O_12_ and Ag_3_PO_4_. As a result, more photogenerated holes and electrons are available for participation in the photocatalytic reactions. The reactive species are determined by investigating the effect of ethanol, BQ and AO on the RhB degradation, and it is concluded that h^+^ is the dominant reactive species and •O_2_^−^ is the secondary reactive species in the present Bi_4_Ti_3_O_12_/Ag_3_PO_4_ photocatalytic system.

## Abbreviations

BET: Brunauer-Emmett-Teller
CB: Conduction band
EIS: Electrochemical impedance spectroscopy
FTO: Fluorine-doped tin oxide
NMP: 1-Methyl-2-pyrrolidione
PL: Photoluminescence
PVDF: Polyvinylidene fluoride
RhB: Rhodamine B
SCE: Standard calomel electrode
SEM: Scanning electron microscopy
TEM: Transmission electron microscopy
TPA: Terephthalic acid
UV-vis DRS: Ultraviolet-visible diffuse reflectance spectroscopy
VB: Valence band
XPS: X-ray photoelectron spectroscopy
XRD: X-ray powder diffraction
